# Fast-track total knee arthroplasty improved clinical and functional outcome in the first 7 days after surgery: a randomized controlled pilot study with 5-year follow-up

**DOI:** 10.1007/s00402-018-3001-2

**Published:** 2018-07-19

**Authors:** Bas L. Fransen, Marco J. M. Hoozemans, Kirsten D. S. Argelo, Lucien C. M. Keijser, Bart J. Burger

**Affiliations:** 1 Department of Orthopaedic Surgery, CORAL-Centre for Orthopaedic Research Alkmaar, Noordwest Ziekenhuisgroep Alkmaar, Wilhelminalaan 12, 1815 JD Alkmaar, The Netherlands; 2 Department of Human Movement Sciences, Faculty of Behavioural and Movement Sciences, Vrije Universiteit Amsterdam, Amsterdam Movement Sciences, Van der Boechorststraat 7, 1081 BT Amsterdam, The Netherlands

**Keywords:** Total knee arthroplasty, Fast-track protocol, Functional outcome

## Abstract

**Introduction:**

Fast-track protocols (FP) are used more and more to optimize results after total knee arthroplasty (TKA). Many studies evaluating FP in TKA concentrate on clinical outcome and medium to long-term results. Since discharge from hospital after TKA is achieved increasingly quicker worldwide using FP in an increasingly younger and active patient population, the effects of FP on functional outcome in the first days after TKA become more important. The purpose of the current study was to compare FP with a regular joint care protocol (RP), with an emphasis on the first 7 days after surgery.

**Materials and methods:**

A non-blinded randomized controlled clinical pilot study was performed with 25 patients assigned to a FP group and 25 patients assigned to a RP group. Primary outcome was functional outcome, clinical outcome, pain, and complications for each day in the first week after surgery. Patients were followed up to 5 years after surgery.

**Results:**

Significantly lower VAS scores for knee pain, faster Timed-Up and Go test times and more mobility on functional tests were seen on several days in the first week in the FP group compared to the RP group. Few other significant differences were found at 2, 6 weeks, and no significant differences were found at 12 weeks and 1, 2 and 5 years after surgery.

**Conclusions:**

Fast-track protocol for primary TKA showed significantly lower knee pain scores and improved functional outcome in the first 7 days after TKA compared to a regular protocol.

## Introduction

Total knee arthroplasty (TKA) has been performed since the 1960s and has significantly improved the quality of life of patients suffering from osteoarthritis of the knee [1, 2]. Recent trends show that patients undergo surgery at a younger age [3, 4]. Furthermore, they want to be able to return to their daily activities and work as soon as possible [3]. To accommodate these trends and to further optimize outcome in TKA patients, patient care is continuously being improved throughout the patient’s hospital admission.

Due to multimodal analgesia, and improvements in wound care, physical therapy, operative techniques and hospital logistics, length of stay (LOS) has decreased to such an extent that 75% of patients remain in hospital for 3 days or less after TKA. A combination of these measures has increasingly been used in fast-track protocols [5]. The first fast-track protocols reduced LOS from an average of 2 weeks to less than 1 week [6]. Since then fast-track protocols for TKA have been shown to reduce LOS even further to maintain patient satisfaction without increasing the number of complications or readmissions [5, 7–10], to reduce morbidity and mortality, to increase cost-effectiveness [11], and to be feasible in all age groups [12, 13].

These studies show that a fast-track protocol leads to similar results in the mid- to long-term follow-up compared to regular protocols. To fully understand the effects of fast-track TKA, studies should also focus on the short-term outcome. A few studies have described short-term results of fast-track TKA and reported good outcomes with respect to pain reduction [7, 14] and the Timed Up and Go (TUG) test [15] in the first days after surgery. Unfortunately, these results were not compared to a non-fast-track control group.

Therefore, the main goal of the present pilot randomized clinical trial was to evaluate whether our fast-track TKA protocol [called the 2 day knee (2DK) protocol] resulted in better outcome than a regular protocol in patients who underwent primary TKA, with an emphasis on the daily clinical and functional outcomes in the first week after surgery. The secondary goal was to compare outcomes between both treatment protocols up to 5 years after surgery.

## Materials and methods

### Study design

A feasibility pilot study was done to provide early indications whether the possible benefits of the 2DK protocol in the first week after surgery were sufficient to justify a long-term study with a larger patient population. A single centre, non-blinded, randomized controlled clinical trial (RCT) was performed. After inclusion and providing informed consent, patients were randomly assigned to either the regular joint care protocol (RP) or the 2DK fast-track protocol (FP), which is used in our hospital, with an allocation ratio of 1:1, after which the baseline measurements were performed. All procedures were in accordance with the Declaration of Helsinki [16], the CONSORT guidelines [17], and Good Clinical Practice guidelines [18]. A Medical Ethical Committee gave approval for this study under number NL33089.094.10. The study protocol was registered in the International Standard Randomised Controlled Trial Number Register with number ISRCTN51839535. When 20 patients had been treated, an interim analysis was performed which showed no contra-indication for continuing the study. After 2 years, the decision was made not to perform a larger scale study since most measures from the protocol had already been implemented in daily practice. However, patients were asked to fill in the questionnaires one additional time 5 years after surgery to determine whether the differences found in the first week had effects on mid-term outcome.

### Study population

Patients were eligible for inclusion if they required a primary unilateral TKA, had American Society of Anaesthesiologists (ASA) status I or II, and were willing and able to comply with the scheduled postoperative clinical and radiographic evaluations and with the rehabilitation program. Patients were excluded if they had other lower limb problems or were diagnosed with insulin-dependent diabetes, severe osteoporosis, rheumatoid arthritis, or a different inflammatory cause for osteoarthritis.

### Intervention and control protocols

All patients in both groups received Scorpio cruciate-retaining total knee prostheses (Stryker, Mahwah, USA). Premedication consisted of paracetamol 1000 mg and temazepam 10 mg. All surgeries were performed by one of two experienced orthopaedic surgeons, each of whom operated patients in both groups. The physical therapy protocol was identical for both groups. Patients were discharged if they were able to ambulate independently—either with two crutches or a walker—and if there were no wound problems. Patients who did not have a good domestic support network, or who did not mobilize adequately according to the physical therapist were referred to a rehabilitation facility. Here they continued training until they were able to return home, which was usually achieved within 1–2 weeks. Thrombo-embolic prophylaxis was fraxiparine 2850 international units once a day for 4 weeks. No steroids were given as part of the protocols. There were no differences between the two groups in the preoperative preparation of patients.

#### Fast-track protocol

In the fast-track protocol (FP), no tourniquet was used during the operation. Omitting the tourniquet was assumed to reduce pain, bleeding and swelling after surgery, thereby leading to a possible faster activation of muscle function and performance. The operation was performed through a subvastus approach, a patella-in-place balancer was used, and patients received intra-operative local infiltration analgesia (LIA) [19]. All patients received a patella component. The risk for infection was minimized by not using pain pumps, wound drains or bladder catheters. The post-operative protocol focused on rapid mobilization under guidance of a physiotherapist. Postoperatively patients received paracetamol 1000 mg four times a day, diclofenac 50 mg three times a day (unless they had an allergy for non-steroidical anti-inflammatory drugs) and oral oxynorm 5 mg only when needed. Patients in the FP were told to expect being discharged from the hospital 2 days after surgery.

#### Regular protocol

The regular protocol (RP) group underwent the regular hospital TKA protocol, which included the use of a tourniquet, wound drains and bladder catheter. The operation was performed through a midline approach. All patients received a patella component. Mobilization was started the first day after surgery, and patients were told beforehand that the average discharge was 4 days after surgery. Similar to the FP, postoperatively patients received identical doses of paracetamol and diclofenac. Contrary to the FP group, patients started with a patient-controlled analgesia (PCA) pump with intravenous morphine. Patients in both groups reduced opioid use as soon as pain allowed this. All differences between the two protocols are shown in Table 1.

**Table 1:** Differences between RP (regular protocol) and FP (fast-track protocol)

Regular protocol (RP)	Fast-track protocol (FP)
Spinal anaesthesia if possible	General anaesthesia
Medial parapatellar approach	Subvastus approach
No patella in-place balancing	Patella in-place balancing
Soft tissue releases if required	No or limited soft tissue releases
Tourniquet	No tourniquet
Patient-controlled analgesia (PCA) with iv morphine, wound drains and bladder catheter	No pain pumps, wound drains or bladder catheter
No local infiltration analgesia (LIA)	LIA intra-operatively
No ice packs	Use of ice packs (3 × 3 times per day)
Special chair to elevate leg and to get out of chair	No special chairs
Joint loading 1 day after surgery	Immediate loading of the joint
Standard short-acting opiates	Short acting opiates only when requested

### First week measurements

Patients were requested to keep a daily diary for the first week after surgery. While in the hospital, the patients were assisted by a nurse and were instructed in how to complete the diary on a daily basis at home. The outcome measures included were the visual analogue scale (VAS) scores for knee pain and the Timed Up and Go test (TUG) [20] scores. The VAS scores ranged from 0 (best) to 100 (worst), and were measured both with the patient resting in bed as well as during movement with weight bearing on the knee. VAS scores in rest were also obtained immediately after surgery in the recovery room, and at 1 and 2 h after surgery. The TUG scores were measured in seconds, a lower score indicating better function. Furthermore, patients were asked to report daily for the first 7 days after surgery whether they were able to perform a straight leg raise, stand on their affected leg for 5 s, walk the stairs independently and/or were able to stand up from a sitting position.

### Short-term and mid-term outcomes

To evaluate short-term outcomes, patients were asked to return to the outpatient clinic at 2, 6 and 12 weeks postoperatively. At these visits, function was assessed using the TUG test (at 2 and 6 weeks postoperatively only) and by assessing the knee range of motion (ROM) measured with a goniometer. Maximum flexion and extension were examined both actively and passively, and extension lag was described with a negative value, while hyperextension was described with a positive value. Clinical outcome was assessed with the VAS knee pain score (both at rest and during movement), the Short Form 12 (SF-12), and the Knee injury and Osteoarthritis Outcome Score (KOOS). The KOOS [21] measures outcome in five subscores, ranging between 0 (worst) and 100 (best). The SF-12 health survey [22] was used to measure quality of life (QoL) in mental and physical subscores, with higher scores indicating a higher QoL. To assess mid-term clinical outcome, patients were asked to complete the KOOS and SF-12 at 1-, 2- and 5-year follow-up. After the 5-year interval, the VAS knee pain score was included in the follow-up measurements.

### Complications

All complications were registered for each group. At each follow-up measurement, the researcher assessed whether a complication had occurred.

### Sample size

Since this was a pilot study, no sample size calculation was performed. In each group, 25 patients were included, which was deemed sufficient to provide an indication about the early results of the fast-track protocol.

### Randomisation and blinding

An independent researcher randomly allocated a protocol (25 RP and 25 FP) to study numbers 1 through 50, and the allocation was concealed in sealed envelopes. After a patient was included and a study number was assigned, a research assistant opened the envelope. Patients were told their allocation before surgery, since it was impossible to keep the patients blinded because of the different incisions and rehabilitation protocols.

### Statistical analysis

Independent *t* tests or their non-parametric equivalent were performed on all patient characteristics and baseline measurements for normally and not-normally distributed data, respectively. After the first week, outcome measurements were analysed using independent *t* tests to compare the means of continuous variables, and Chi-squared tests for categorical variables, using Fisher’s exact test when applicable. The short- and mid-term data were analysed by calculating the differences between the baseline measurement and each follow-up measurement (Δ-score). Independent *t* tests were performed on the Δ-scores. All analyses were performed with IBM SPSS Statistics version 20 (IBM Corporation, Armonk, NY, USA). A *p* value of *p* < 0.05 indicated statistical significance. Calculations were done for each follow-up measurement for all patients not lost to follow-up until that point in time.

## Results

### Study population

**Fig. 1:**
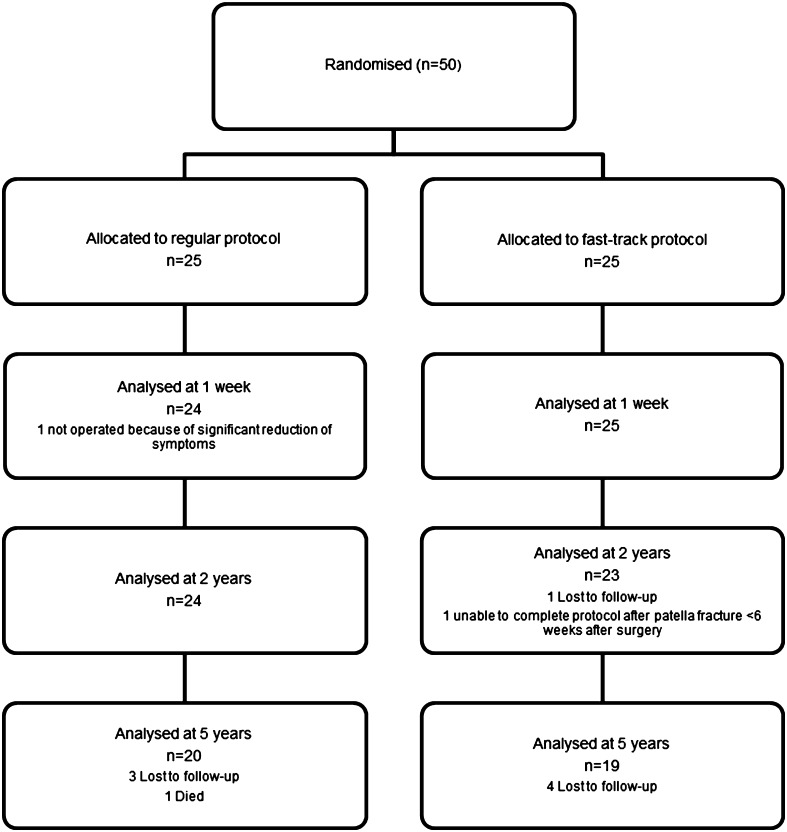
Diagram with participant flow. 20/25 RP patients and 19/25 FP patients completed the total 5-year follow-up measurements

The study took place between May 2011 and June 2017 at the orthopaedic department of a large-volume teaching hospital in the Netherlands. Out of 50 included patients, one patient in the RP group did not undergo surgery because of a significant reduction in symptoms. Not all patients completed every question in the diary during the first 7 days. The entire 5-year protocol was completed by 39 patients, 19 in the FP group and 20 in the RP group (Fig. 1).

### Patient characteristics and baseline measurements

Patient groups were comparable at baseline (Table 2). Length of hospital stay (LOS) was significantly shorter for patients in the FP group [mean (SD) 3.7 (1.8) days vs 4.7 (1.3) days, *p* = 0.036]. Duration of the surgery was significantly longer and intra-operative blood loss was higher for the FP group compared to the RP group. Four patients were discharged to a rehabilitation facility, all part of the RP group.

**Table 2:** Patient characteristics and baseline measurements

Scores	Baseline		
Mean (SD) or *N* (%)	RP (*N* = 24)	FP (*N* = 25)	*p* value
Age (years) at baseline	61 (7)	64 (9)	0.165
BMI (kg/m^2^) at baseline	30.0 (4.1)	28.7 (3.5)	0.254
Duration of surgery (minutes)	76.5 (15.3)	102.1 (20.4)	< 0.001
Blood loss (ml) during surgery	45.8 (127.6)	261.0 (200.8)	< 0.001
Length of hospital stay (days)	4.7 (1.3)	3.7 (1.8)	0.036
Gender			
Female	15 (63%)	14 (56%)	0.644
Male	9 (37%)	11 (44%)	
Affected knee			0.674
Left	12 (50%)	11 (44%)	
Right	12 (50%)	14 (56%)	
Type of anaesthesia			0.235
General	22 (92%)	25 (100%)	
Spinal	2 (8%)	0	

No significant differences were found between the FP and RP groups in the preoperative measurements. Duration of surgery and blood loss were significantly higher in the FP group. LOS was shorter in the FP group

*SD* standard deviation, *RP* regular protocol, *FP* fast-track protocol, *BMI* body mass index

### First week measurements

**Fig. 2:**
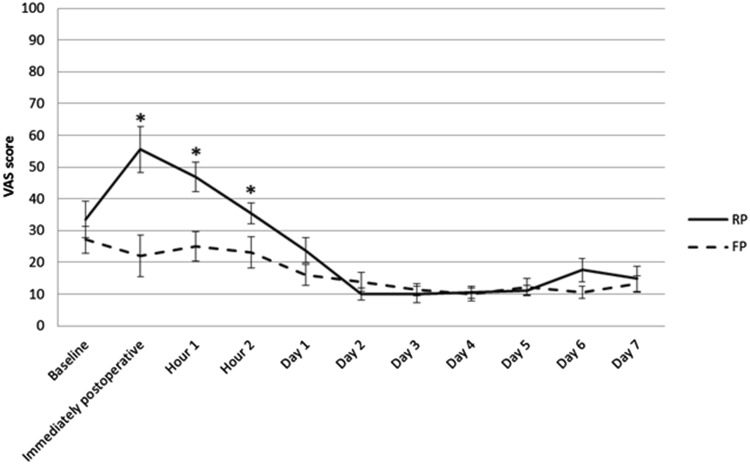
First-week VAS scores for knee pain at rest. Mean VAS scores for knee pain in rest of the RP and FP groups in the first 7 days, with error bars showing standard error of the mean. Significantly lower scores in the FP group were found immediately postoperative, 1 and 2 h after surgery

**Fig. 3:**
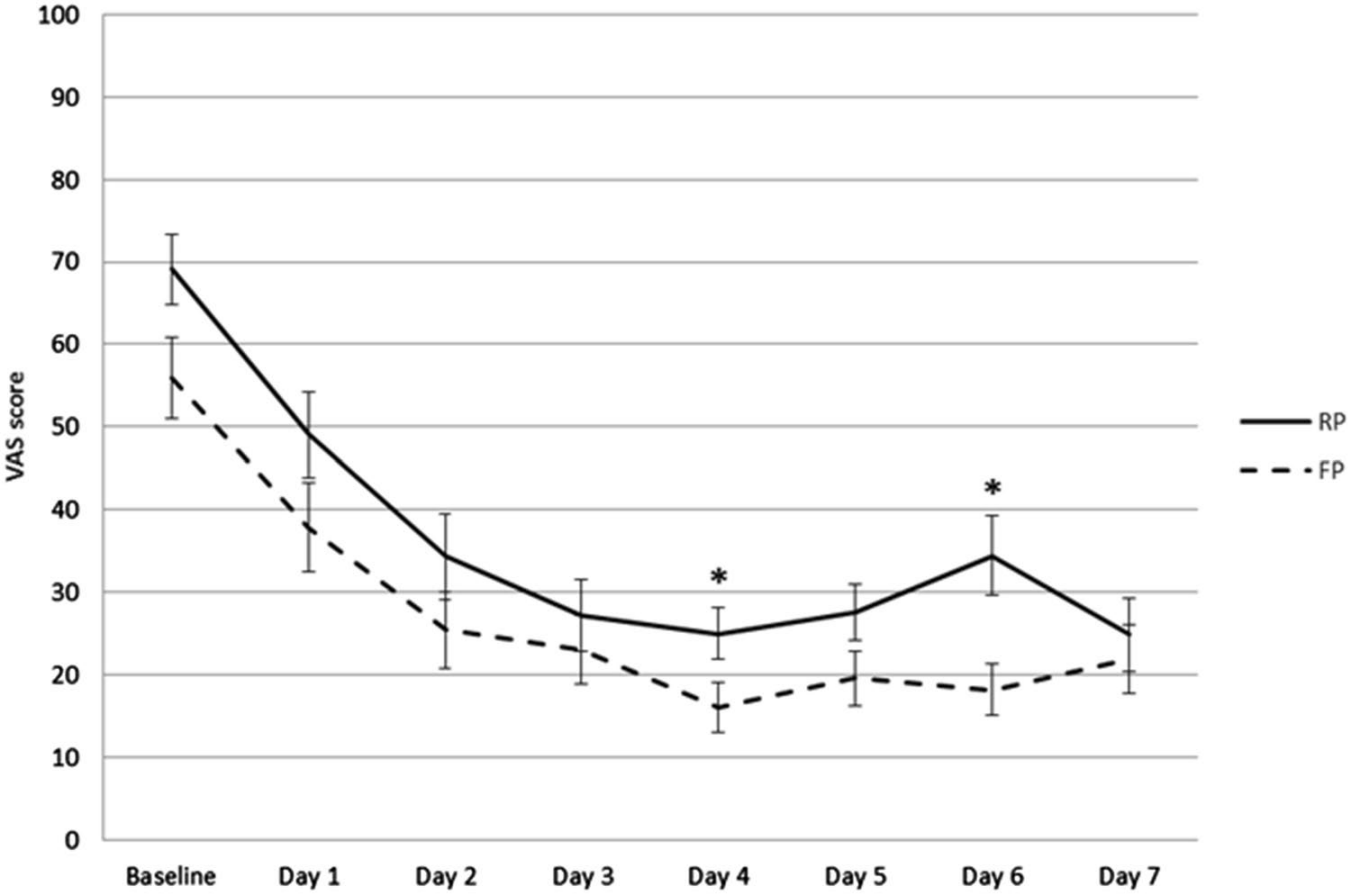
First-week VAS scores for knee pain during movement. Mean VAS scores for knee pain during movement with weight bearing of the affected knee of the FP and RP groups in the first 7 days, with error bars showing standard error of the mean. Scores were lower in the FP group on all days, with significant differences on the fourth and sixth days after surgery

**Fig. 4:**
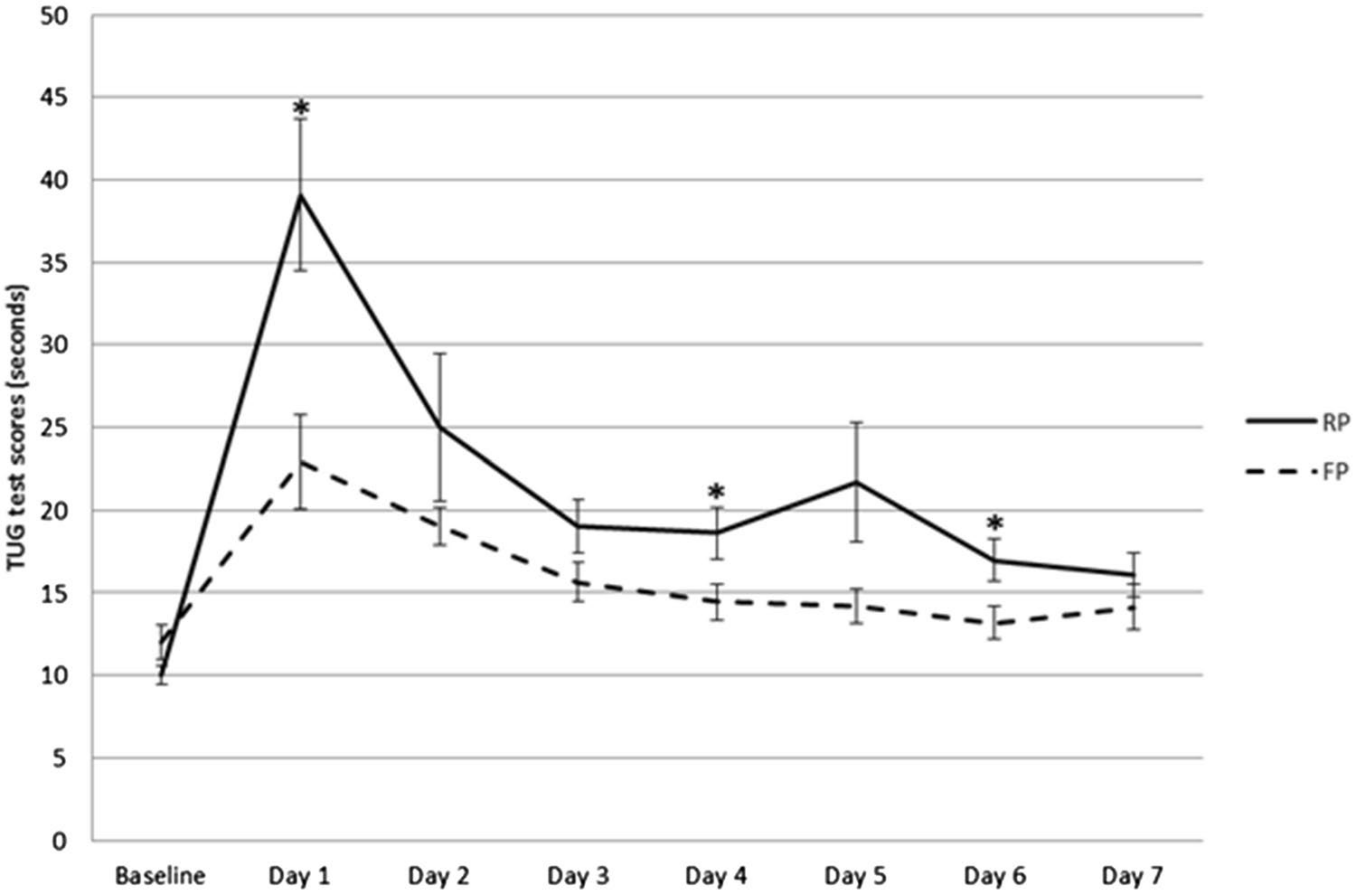
First-week TUG test scores in seconds. Mean TUG test scores in seconds of the FP and RP groups in the first 7 days, with error bars showing standard error of the mean. On the fourth and sixth day postoperative, the FP group was significantly faster compared to the RP group

VAS scores for knee pain at rest were significantly lower in the FP group compared to the RP group immediately postoperative [mean (SD) RP 56 (33) vs FP 22 (31), *p* < 0.001], at 1 h after surgery [RP 47 (23) vs FP 25 (22), *p* = 0.002], and at 2 h after surgery [RP 36 (14) vs FP 23 (18), *p* = 0.035] (Fig. 2). There were no significant differences in VAS scores for knee pain at rest for the next 7 days. The VAS scores during movement were consistently lower in the FP group, with significant differences at 4 days after surgery [mean (SD) RP 25 (16) vs FP 16 (13), *p* = 0.048] and at 6 days after surgery [RP 34 (24) vs FP 18 (14), *p* = 0.007] (Fig. 3). Patients in the FP group had better scores on the TUG test throughout the first week, with statistically significant differences after 1 day [mean (SD) RP 39 (15) vs FP 23 (10), *p* = 0.008], 4 days [RP 19 (8) vs FP 14 (5), *p* = 0.037] and 6 days [RP 17 (6) vs FP 13 (5), *p* = 0.029] (Fig. 4).

The results of the functional questions during the first week are listed in Table 3. Significantly more patients in the FP group were able to stand on their operated leg for 5 s at day one, day two and day six. On the first day, significantly more patients in the FP group were able to do a straight leg raise. Stair climbing was not possible for any of the RP patients on the first day, compared to five patients in the FP group, which was a significant difference. No significant differences were found in the other first-week diary outcomes.

**Table 3:** First week functional test scores

Scores	Day 1			Day 2			Day 3			Day 4		
Number (%)	RP	FP	*p* value	RP	FP	*p* value	RP	FP	*p* value	RP	FP	*p* value
Stretched leg raise			< 0.001			0.357			0.648			0.362
Yes	7 (28%)	21 (95%)		21 (84%)	20 (95%)		23 (92%)	18 (86%)		21 (84%)	19 (95%)	
No	18 (72%)	1 (5%)		4 (16%)	1 (5%)		2 (8%)	3 (14%)		4 (16%)	1 (5%)	
Rise from chair			0.414			1			0.561			0.534
Yes, without help	4 (17%)	7 (33%)		11 (44%)	9 (43%)		13 (52%)	13 (62%)		14 (58%)	14 (70%)	
Yes, with help	18 (75%)	13 (62%)		14 (56%)	12 (57%)		12 (48%)	8 (38%)		10 (42%)	6 (30%)	
No	2 (8%)	1 (5%)		0	0		0	0		0	0	
Stair climbing			0.02			0.841			0.309			0.215
Yes, normal	0	1 (6%)		1 (4%)	1 (4%)		3 (13%)	1 (4%)		3 (13%)	1 (5%)	
Normal up, down with handrail	0	0		3 (12.5%)	1 (4%)		1 (4%)	2 (10%)		1 (4%)	5 (25%)	
Up and down with handrail	0	4 (22%)		17 (71%)	16 (76%)		20 (83%)	16 (76%)		19 (79%)	13 (65%)	
Not possible	25 (100%)	13 (72%)		3 (12.5%)	3 (16%)		0	2 (10%)		1 (4%)	1 (5%)	
5-s leg stand			0.003			0.03			0.297			0.056
Yes	3 (12%)	11 (58%)		15 (60%)	17 (89%)		17 (68%)	17 (85%)		17 (68%)	18 (95%)	
No	21 (88%)	8 (42%)		10 (40%)	2 (11%)		8 (32%)	3 (15%)		8 (32%)	1 (5%)	

Scores	Day 5			Day 6			Day 7		
Number (%)	RP	FP	*p* value	RP	FP	*p* value	RP	FP	*p* value
Stretched leg raise			1			0.198			0.608
Yes	22 (88%)	19 (91%)		20 (80%)	20 (95%)		20 (87%)	21 (95%)	
No	3 (12%)	2 (9%)		5 (20%)	1 (5%)		3 (13%)	1 (5%)	
Rise from chair			0.522			0.325			1
Yes, without help	16 (64%)	16 (76%)		16 (64%)	17 (81%)		18 (78%)	17 (77%)	
Yes, with help	9 (36%)	5 (24%)		9 (36%)	4 (19%)		5 (22%)	5 (23%)	
No	0	0		0	0		0	0	
Stair climbing			0.683			0.464			0.538
Yes, normal	3 (12%)	3 (15%)		2 (8%)	3 (14%)		2 (9%)	3 (14%)	
Normal up, down with handrail	5 (20%)	3 (15%)		4 (16%)	5 (24%)		4 (18%)	6 (27%)	
Up and down with handrail	17 (68%)	13 (65%)		19 (76%)	12 (57%)		16 (73%)	12 (55%)	
Not possible	0	1 (5%)		0	1 (5%)		0	1 (4%)	
5-s leg stand			0.26			0.027			0.699
Yes	18 (72%)	18 (90%)		17 (68%)	20 (95%)		18 (78%)	19 (86%)	
No	7 (28%)	2 (10%)		8 (32%)	1 (5%)		5 (22%)	3 (14%)	

Numbers are shown of the number of patients in the RP and FP groups reporting their ability to perform a stretched leg raise, rise from a chair, climb stairs or stand on their affected leg. A significantly higher number of patients in the FP group was able to stand on their operated leg for 5 s on day one, two and six and were able to climb the stairs and do a straight leg raise on day one

*RP* regular protocol, *FP* fast-track protocol

### Short and mid-term outcome

A significant difference was found 2 weeks after surgery in the TUG Δ-scores [mean (SD) RP 2.4 (3.3) s vs FP − 0.4 (4.4), *p* = 0.017], with the RP group showing an increase in TUG times compared to a decrease in the FP group (Table 4). There was a significant difference between the groups in the SF-12 physical score at 6 weeks, with the RP group showing more improvement than the FP group [mean (SD) RP 6.0 (8.1) vs FP 0.4 (7.1), *p* = 0.021]. The VAS scores for pain during knee movement at 6 weeks were significantly more reduced compared to baseline in the RP group than in the FP group [mean (SD) RP − 51.7 (24.7) vs FP − 33.8 (30.3), *p* = 0.031]. No significant differences between groups were seen after 12 weeks in both clinical and functional outcome parameters, or in any of the clinical outcome parameters at 1, 2 and 5 years after surgery (Table 5).

**Table 4:** Short-term outcome parameters

Δ-scores	2 weeks			6 weeks			12 weeks		
	RP	FP	*p* value	RP	FP	*p* value	RP	FP	*p* value
	Mean (SD)	Mean (SD)		Mean (SD)	Mean (SD)		Mean (SD)	Mean (SD)	
SF-12									
Physical score	− 0.2 (4.6)	− 0.2 (4.5)	0.973	6.0 (8.1)	0.4 (7.1)	0.021	7.4 (8.5)	5.3 (7.5)	0.397
Mental score	0.0 (4.6)	0.6 (8.0)	0.784	− 2.4 (6.1)	1.0 (8.1)	0.139	− 0.7 (5.6)	− 0.6 (5.8)	0.965
KOOS									
Symptom score	5.3 (17.4)	4.2 (24.5)	0.848	7.0 (23.2)	2.1 (22.5)	0.457	16.9 (18.0)	10.9 (26.1)	0.362
Pain score	18.7 (19.4)	15.2 (19.1)	0.532	21.3 (19.5)	12.7 (21.5)	0.151	29.0 (23.3)	26.2 (24.8)	0.693
ADL score	18.1 (21.9)	13.7 (22.3)	0.49	28.7 (24.7)	15.0 (27.0)	0.073	29.5 (23.0)	22.4 (26.1)	0.324
Sport and recreation score	0.0 (17.8)	− 1.0 (21.4)	0.86	6.7 (25.1)	6.9 (23.4)	0.976	21.0 (30.2)	14.8 (24.2)	0.438
QoL score	15.4 (20.3)	19.5 (26.3)	0.549	24.9 (19.3)	20.8 (25.2)	0.527	27.8 (23.0)	34.3 (29.0)	0.395
VAS									
Movement	− 46.1 (24.4)	− 38.6 (30.8)	0.358	− 51.7 (24.7)	− 33.8 (30.3)	0.031	− 48.4 (26.5)	− 37.3 (29.7)	0.184
Rest	− 18.5 (35.3)	− 15.0 (27.7)	0.705	− 23.3 (27.7)	− 14.0 (26.7)	0.250	− 20.5 (27.8)	− 21.1 (23.7)	0.943
TUG (s)	2.4 (3.3)	− 0.4 (4.4)	0.017	− 0.2 (3.0)	− 1.7 (3.3)	0.116			
ROM (°)									
Passive									
Flexion	− 19 (14)	− 19 (15)	0.954	− 7 (14)	− 10 (18)	0.495	1 (16)	− 3 (15)	0.45
Extension	− 2 (6)	− 4 (7)	0.234	0 (6)	− 1 (4)	0.357	1 (6)	1 (6)	0.868
Active									
Flexion	− 20 (14)	− 22 (16)	0.58	− 9 (13)	− 13 (18)	0.376	0 (16)	− 3 (15)	0.428
Extension	− 3 (7)	− 5 (6)	0.189	− 1 (6)	− 1 (5)	0.917	1 (6)	1 (6)	0.981

Mean change (Δ-scores) for the SF-12, KOOS, VAS for knee pain, TUG and knee ROM are shown at 2, 6 and 12 weeks postoperatively. TUG test times were faster in the FP group after 2 weeks. SF-12 and VAS for knee pain during movement scores had improved more in the RP group after 6 weeks. Data in mean (standard deviation)

*Δ-scores* score at follow-up moment minus baseline score, *KOOS* Knee Injury and Osteoarthritis Outcome Score, *RP* regular protocol, *FP* fast-track protocol, *ADL* activities of daily living

**Table 5:** Mid-term outcome parameters

Δ-scores	1 year			2 years			5 years		
	RP	FP	*p* value	RP	FP	*p* value	RP	FP	*p* value
	Mean (SD)	Mean (SD)		Mean (SD)	Mean (SD)		Mean (SD)	Mean (SD)	
SF-12									
Physical score	9.4 (12.8)	4.9 (9.3)	0.243	6.2 (16.3)	6.0 (10.5)	0.969	9.4 (13.7)	7.4 (9.6)	0.689
Mental score	7.1 (7.1)	6.2 (9.0)	0.751	7.0 (8.1)	8.8 (6.2)	0.546	7.6 (9.2)	6.1 (8.6)	0.645
KOOS									
Symptom score	33.7 (19.1)	22.9 (31.4)	0.183	30.1 (18.3)	35.7 (23.4)	0.429	41.1 (16.9)	37.0 (23.9)	0.566
Pain score	45.0 (19.8)	34.3 (29.3)	0.177	41.3 (21.9)	40.8 (25.9)	0.95	34.3 (22.4)	27.0 (30.6)	0.409
ADL score	41.6 (18.8)	28.5 (27.6)	0.091	37.9 (25.3)	35.1 (21.3)	0.739	33.6 (21.2)	28.1 (24.5)	0.463
Sport and recreation score	42.9 (28.8)	31.0 (32.3)	0.215	36.5 (35.6)	36.9 (32.0)	0.973	47.0 (27.0)	38.1 (27.0)	0.334
QoL score	40.7 (19.6)	39.7 (30.3)	0.896	40.1 (24.4)	45.1 (26.7)	0.567	44.2 (22.4)	46.0 (28.4)	0.830
VAS knee pain									
Movement							− 54.3 (26.0)	− 43.5 (33.5)	0.315
Rest							− 21.0 (18.1)	− 20.9 (23.8)	0.986

This table shows mean change (Δ-scores) for the SF-12, KOOS and VAS for knee pain for the 1, 2 and 5 years follow-up measurements. No significant differences between the RP and FP groups were found. Data in mean (standard deviation)

*Δ-scores* score at follow-up moment minus baseline score, *KOOS* Knee Injury and Osteoarthritis Outcome Score, *RP* regular protocol, *FP* fast-track protocol, *ADL* activities of daily living

### Complications

All complications are described in Table 6. The number and severity of the complications in both groups were comparable. One patient in the RP group underwent revision surgery (replacement of insert) because of persisting instability of the knee. In both groups, two patients needed manipulation of the knee because of impairment in ROM.

**Table 6:** Complications

Regular protocol		Fast-track protocol	
Complication	Time after surgery	Complication	Time after surgery
Manipulation (2×)	3 and 4 months	Manipulation (2×)	3 and 4 months
Revision surgery (new insert because of instability)	1 year	Pain on the lateral side of knee, treated with injection (2×)	10 months
Limited knee extension, treated with cast	10 weeks	Fractured patella after fall	1.5 years
Swelling, treated with intra-articular injection	1.5 years	Patellar instability, treated with brace	1 year
Urinary tract infection	Directly postoperative		
Meralgia paraesthetica	Directly postoperative		

The number and severity of the complications observed were comparable for the RP and FP groups

## Discussion

We performed a randomized controlled pilot study to evaluate functional and clinical outcomes in the first 7 days after surgery of patients who underwent TKA using either a regular protocol or a fast-track protocol. The study population was followed up to 5 years after their operation. Significant better scores on several functional outcome measures in the first week were seen in the FP group in this study compared to the RP group. This is in line with studies that showed that fast-track protocols showed improvements in reduction of LOS, complications [5], and even a reduction in 30 and 90 day mortality [23]. These improvements have been associated with high patient satisfaction [7].

Comparing studies that analyse fast-track protocols is challenging, since each protocol differs to a smaller or larger extent (e.g. in approach [24], use of analgesics [15], or mobilization schedule [6]). Since fast-track protocols are aimed at starting rehabilitation sooner and mobilizing patients more quickly, this study placed more emphasis on short-term outcome, specifically the first 7 days postoperatively. Our outcomes are in line with those of a few other studies that examined the effects of fast-track TKA on outcome in the first days after the procedure. Two studies, by van Egmond et al. and Winther et al. [7, 14], showed reduction in pain scores in the first few days after fast-track TKA, but did not compare this with a regular protocol. This reduction remained visible after 6 weeks and 1 year, respectively. In the current study, the VAS scores for knee pain at rest were significantly lower in the FP group during the first 2 h after surgery compared with the RP group. In addition, the VAS knee pain scores for pain during knee movement were significantly lower on days four and six in the FP group. Since patients in the FP group were given fewer short-acting opiates, this reduction in pain appears to be mostly due to the combination of omitting the tourniquet, LIA and the use of ice packs. Spinal anaesthesia could have influenced the pain scores in the hours after surgery, but only two patients in the RP group and no patients in the FP group had been given spinal anaesthesia. It is therefore unlikely that this has influenced pain scores. A significant difference in duration of surgery was found with a longer duration in the FP group, which can largely be attributed to the use of the patella-in-place balancer.

When looking at functional results within the first week, the TUG test times were significantly better in the FP group on days one, four, and six. When comparing this to the literature, one study by Holm et al. also reported good results for the TUG test in 100 patients on the first days after TKA surgery with a fast-track protocol [15], although this was not a controlled study. In their study, the median TUG test time at discharge (3 days postoperative) was 19.2 s (interquartile range 25–75% 15.3–24.1), which is comparable to the TUG test times in the patients presented in the present study 3 days after surgery [mean (SD) RP 19 (8) s vs FP 16 (5), *p* = 0.109].

For the other functional parameters assessed with diary questions in the first 7 days, significantly more patients in the FP group were able to stand on their operated leg for 5 s on day one, two and six and were able to climb the stairs and do a straight leg raise on day one in our study. These better results in the FP group could be attributed to the subvastus approach, which together with the patella in place balancing with no or limited soft tissue releases, could lead to the lower knee pain scores seen in the first week. When assessing these diary results, it should be noted that not all patients fully completed the questions on every day, which might have influenced these results even though patients in both groups had missing entries. However, since functional outcome has a large impact on patient satisfaction [25, 26], the signs of better function found in this study in the first days after TKA when using a fast-track protocol confirm the positive effects previously observed on outcome and LOS in fast-track TKA patients.

Functional and clinical short-term outcomes were measured up to 12 weeks after surgery. At 2-week follow-up, patients in the FP group had improved more in the TUG test, but this effect disappeared after 6 weeks. This could indicate that the FP group functioned better after 2 weeks, but the other functional outcome parameters did not show similar effects. Change in ROM of the knee did not differ between the RP and FP groups. It is known that pre-operative ROM influences both LOS [27] and post-operative ROM [28]. However, since pre-operative ROM did not differ between groups, this cannot have biased the postoperative results. TKA has been known to positively effect QoL of patients with osteoarthritis of the knee [29], although this has not been extensively described for short-term follow-up.

The SF-12 physical score after 6 weeks showed a significantly stronger improvement in the RP group than in the FP group. This could have been due to more post-operative pain, since the FP group received less pain medication as part of their protocol. However, this difference was not found after 2 weeks, and the VAS scores for knee pain in rest and during knee movement also did not differ significantly between the two groups after 6 weeks.

In both groups, patients scored at a level deemed satisfactory after 12 weeks [30] for the KOOS, SF-12 and VAS knee pain scores. This is contrary to other studies, which found a positive effect on clinical outcome when using a fast track protocol [6, 7] compared to a regular protocol. However, these studies used a different outcome score (Knee Society Score), which might partly explain why different results were reported. At mid-term follow-up after 1 and 2 years, no significant differences were found in function, pain and QoL, which confirms the results reported by earlier studies [7]. This same pattern was found 5 years after surgery in the KOOS, SF-12 and VAS knee pain scores. To our knowledge, at the time of writing, there were no comparable studies that presented 5-year follow-up data of fast-track compared to regular TKA; hence no comparison to the literature could be made.

A fast-track protocol usually contains several parts that are aimed at improving outcome. For studies evaluating fast-track protocols in general, but especially for a smaller study population as in the current study, it is difficult to determine which parts of the fast-track protocol do or do not contribute to the observed effect on outcome. To determine for each individual element of the fast-track protocol whether it contributes to the outcome, a large number of studies are required. Since a possible contribution would probably have a small effect, large numbers of included patients will be needed. It is therefore also possible that several modifications from the fast-track protocol did not contribute to the positive results found in this study. Furthermore, several hospitals are already experimenting with a next step in fast-track protocol: outpatient TKA surgery. Even though this is relatively new, there have been some signs showing that the experiences gained in fast-track TKA surgery are being used to help patients return home even earlier [31].

One outcome measurement that was not studied but could be of interest is cost-effectiveness. There are studies showing that fast-track protocols are cost-effective in TKA and total hip arthroplasty [32]. This could be due to several factors. First, fast-track protocols could reduce costs because of a reduction in LOS. If all patients left the hospital 1–2 days earlier than usual, regular costs of hospital admittance would decrease. In addition, a longer LOS is associated with an increased use of hospital resources [33]. Second, fast-track protocols could result in better cost-effectiveness because patients that have better function when discharged from hospital would require less pain medication, a lower number of physiotherapy treatments and a lower number of days spent in rehabilitation centres, and they would be able to return to work sooner [34, 35].

### Limitations

There are several limitations to this study. Comorbidities have not been accounted for, even though patients’ comorbidities can influence outcome after TKA [36, 37]. This issue has partly been addressed by only including ASA I and II patients. Similarly, a patient’s perception of his/her hospital stay has not been taken into account, even though this has been shown to influence patient functional outcome and satisfaction [38]. We aimed to tackle both these issues using a randomized allocation of subjects to the study groups. As described, patients were told before surgery that the aim of the FP was to discharge patients after 2 days, which means that it is possible that the LOS outcome was influenced by motivational bias, since patients were focused from the beginning on an early discharge.

## Conclusions

This randomized controlled pilot study comparing the early results of primary TKA patients in a fast-track TKA protocol and a regular protocol showed indications that even with the significantly quicker discharge from hospital associated with fast-track TKA, patients in the fast-track group had lower knee pain scores and better functional outcome in the first 7 days after surgery. Since this only a pilot study, no firm conclusions can be drawn. However, since fast-track protocols for TKA are being implemented in most orthopaedic practices, more emphasis of research on the first days after TKA may provide more opportunities for further improving outcome in fast-track TKA patients.
